# Limited Sequence Diversity Within a Population Supports Prebiotic RNA Reproduction

**DOI:** 10.3390/life9010020

**Published:** 2019-02-21

**Authors:** Ryo Mizuuchi, Niles Lehman

**Affiliations:** Department of Chemistry, Portland State University, Portland, OR 97207, USA; niles@pdx.edu

**Keywords:** origins of life, RNA, reproduction, autocatalytic networks, recombination, ligation

## Abstract

The origins of life require the emergence of informational polymers capable of reproduction. In the RNA world on the primordial Earth, reproducible RNA molecules would have arisen from a mixture of compositionally biased, poorly available, short RNA sequences in prebiotic environments. However, it remains unclear what level of sequence diversity within a small subset of population is required to initiate RNA reproduction by prebiotic mechanisms. Here, using a simulation for template-directed recombination and ligation, we explore the effect of sequence diversity in a given population for the onset of RNA reproduction. We show that RNA reproduction is improbable in low and high diversity of finite populations; however, it could robustly occur in an intermediate sequence diversity. The intermediate range broadens toward higher diversity as population size increases. We also found that emergent reproducible RNAs likely form autocatalytic networks and collectively reproduce by catalyzing the formation of each other, allowing the expansion of information capacity. These results highlight the potential of abiotic RNAs, neither abundant nor diverse, to kick-start autocatalytic reproduction through spontaneous network formation.

## 1. Introduction

Reproduction of information-carrying polymers is integral to the origins of life. In prebiotic environments on the early Earth, reproducing molecules must have originated from limited resources because their supply solely relied on geological factors and inherent chemistry, without the help of complex biological catalysts [1]. Limited types and abundance of biologically relevant molecules found in extraterrestrial objects [2] also imply constrained chemical processes in the solar system. Therefore, the extent of chemical complexity needed to initiate molecular reproduction is a critical parameter for the onset of the living process on the primordial Earth and elsewhere in the universe. 

As envisioned in the RNA world theory, a promising candidate for a prebiotic informational polymer on the early Earth is ribonucleic acid, because it can possess catalytic function, potentially even reproducing itself [3,4]. Although possible prebiotic synthesis and maintenance of building blocks and RNA polymers have been demonstrated [5,6,7,8], the availability of RNAs on the early Earth should have been restricted in several ways. First, the composition of abiotically synthesized RNAs would not be fully random but instead biased; because each nucleotide synthesis prefers different chemical conditions, they degrade differentially, and the different monomers polymerize to form RNA in different rates [9]. Second, the length of RNAs produced by prebiotic polymerization is significantly biased to short ones [5,10,11], making the advent of a complex ribozyme such as polymerase ribozyme [12] unlikely. Third, introduction of compartments or equivalent spatial structures, likely requisites for sustainable propagation of genetic information [13,14], also limits the availability and variety of RNAs.

Pertaining to such abiotic RNA oligomers, one possible reproduction mechanism is template-directed recombination [15,16]: A phosphodiester exchange reaction that shuffles two sequences to synthesize a new set of different lengths (e.g., 8-mer + 8-mer → 12-mer + 4-mer). These reactions are nearly energy-neutral but potentially irreversible if driven by the formation of stable secondary structures, and hence are prebiotically facile. Recombination of RNA oligomers has been recently demonstrated to occur readily, by a number of mechanisms [17,18]. The products could serve as a template for reactions that in turn catalyze their own production (i.e., autocatalytic reproduction). We defined this type of reaction that produces a new very similar (or the same, as analyzed in this study) RNA sequence to a template as “reproduction” to distinguish it from a typical “replication” reaction that produces a complementary RNA strand by polymerization chemistry. Another possible reaction for reproduction is template-directed ligation, which conjugates two sequences (e.g., 8-mer + 8-mer → 16-mer). Although template-directed ligation has been extensively examined [9,17,19,20], the availability of required pre-activated fragments in prebiotic conditions is arguable.

A plausible organization of prebiotic reproduction is an autocatalytic network, in which each molecule multiplies one of the others, allowing collective reproduction [21], as supported by both theoretical [22,23,24,25,26,27] and experimental research [19,28,29]. Indeed, extensive theoretical analysis on a well-known binary polymer model of catalytic reaction showed that such an autocatalytic network inevitably exists and reproduction occurs, provided that all the possible molecules up to a certain polymer length are simultaneously and infinitely available [22,24]. This was true as well in the case of a template-directed ligation [26]. However, whether a set of reproducing polymers such as RNAs can arise from a small subset of finite molecules is unclear. 

Here, using computer simulations, we investigated the required diversity of finite populations of short (8-mer) RNAs for the onset of autocatalytic reproduction under prebiotically possible catalytic reactions. We mainly focused on template-directed recombination, but we also compared it to template-directed ligation. We found that in both cases, a high diversity of an RNA population was not required to initiate reproduction; in fact, it rather diminished the chance of reproduction in finite populations. The reproduction at high diversity was improved to some extent with increased population size. Conversely, extremely limited variation of RNA sequences also made reproduction unlikely. We discovered that the critical sequence diversity within a population for the initiation of reproduction is in an intermediate range, and in that range, autocatalytic networks were favorably formed with a high probability.

## 2. Materials and Methods

### 2.1. Model Description

In a simulation, performed in the Python 3.6 environment on a PC, supplied RNA sequences randomly reacted with each other according to a template-directed recombination or a template-directed ligation mechanism as described below. We defined *X* as distinct 8-mer sequences (i.e., initial sequence diversity in a given population), and prepared a fixed number of each *X* distinct sequence (e.g., 300 copies of *X* distinct sequences) as an initial population. Each distinct 8-mer sequence was randomly sampled from all of the possible combination of 4 nucleotide variants, assuming adenine (A), guanine (G), cytosine (C), and uracil (U), as seen in RNA. Assumed from physiological RNA chemistry, A can form a base-pair with U, and G with C. During one reaction step, two sequences were randomly picked up as potential substrates from the population, and another sequence was also picked up as a potential template, and then template-directed recombination or template-directed ligation occurred if they satisfied criteria described below. 

**Figure 1:**
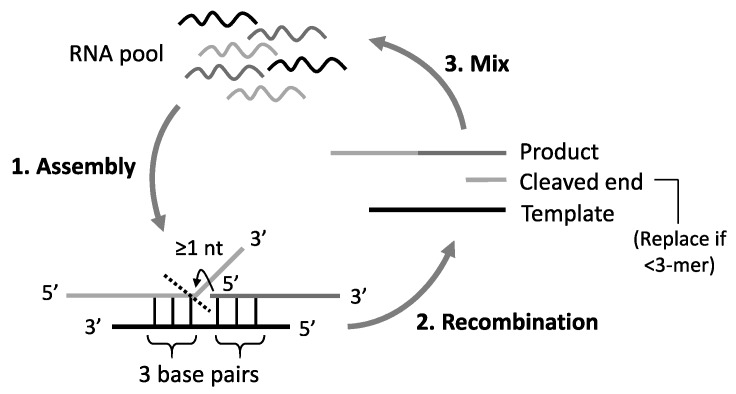
Schematic representation of the simulation under template-dependent recombination. (1) One template and two substrate RNAs were randomly chosen from an RNA population, and they assembled to form base pairs between the substrates and the template. (2) The substrates recombined if the complex satisfied the criteria described in the main text: E.g., three consecutive base pairs on either side of the recombination junction. The left-side substrate was cleaved at the nucleotide indicated by the dashed line and ligated with the right-side substrate. One recombination reaction generated a product, a cleaved end, and the template. (3) They were put back into the RNA population or replaced with an RNA in the initial population if their lengths were too short to react (<3-mer). This cycle was repeated up to a given number of reaction steps.

Template-directed recombination occurs as a combination of cleavage of one substrate RNA and subsequent ligation of it to the other substrate RNA, supported by a template RNA that forms base-pairs with the two substrates [15,18]. We assumed the reaction was catalyzed by a template oligomer if the duplex formed six consecutive base pairs, three with the 5′ end of one RNA substrate and the other three with any part of the other substrate except for the 3′ end single nucleotide (Figure 1). More specifically, we first let the 5′ end of one of the RNA substrates form three consecutive base pairs with any part of the template, and then we let any part of the other substrate except for the 3′ end single nucleotide form another three consecutive base pairs with the template at the immediately downstream of the 3′ end of the base-paired region. If there were multiple possible base pairings in this process, one of them was randomly chosen. For template-directed ligation, we assumed the reaction was catalyzed by a template oligomer if it formed six consecutive base pairs, three with the 5′ end of one RNA substrate and the other three with the 3′ end of the other substrate. We assumed the three base pairs on either side of the reaction junctions was sufficient for a reaction, as demonstrated in oligonucleotide reproduction based on a template-directed ligation [19]. After this step, reacted and unreacted sequences were put back into the population. 

We fixed the concentration of reactable RNAs in a population over iterative reactions so that the time of a reaction could be assumed the same, and that the population size did not change. To do this, we added one molecule chosen randomly from an initial population if ligation occurred, or substituted the molecule for an unreactable short RNA (<3-mer) generated by recombination. For simplicity, degradation of RNAs by hydrolysis was not considered because we did not have sufficient empirical data of relative hydrolysis rate to the rate of the template-directed reactions. We also did not consider template-inhibition for the subsequent reaction, a potential problem for prebiotic reactions, although they could be overcome in fluctuating environments [30]. The sequence of reactions was repeated up to a given number of reaction steps, proportional to population size for each simulation (Figure 1).

### 2.2. Graph Analysis of Simulation

In a simulation, every pair of catalysts (template) and products (ligated oligomers) for every reaction was recorded to create a directed graph. Only the products that remained in the final population of a simulation was considered. To remove randomness, only the reactions that occurred more than once were considered. The directed graph was created by the Python software package NetworkX. Closed catalytic reaction sets, including the self-reproduction reaction, was obtained using the same package. To estimate the maximum size of a closed autocatalytic network, the sizes of as many as possible closed networks were calculated, also using the same package. Because the calculation time exponentially increases as the number of edges involved in a network increases, we set 2.0 s for the maximum calculation time, which was long enough for a calculated maximum size to be saturated in our simulation, although calculation time was less than 0.2 s in most cases.

## 3. Results

### 3.1. Directed Graph Representations

**Figure 2:**
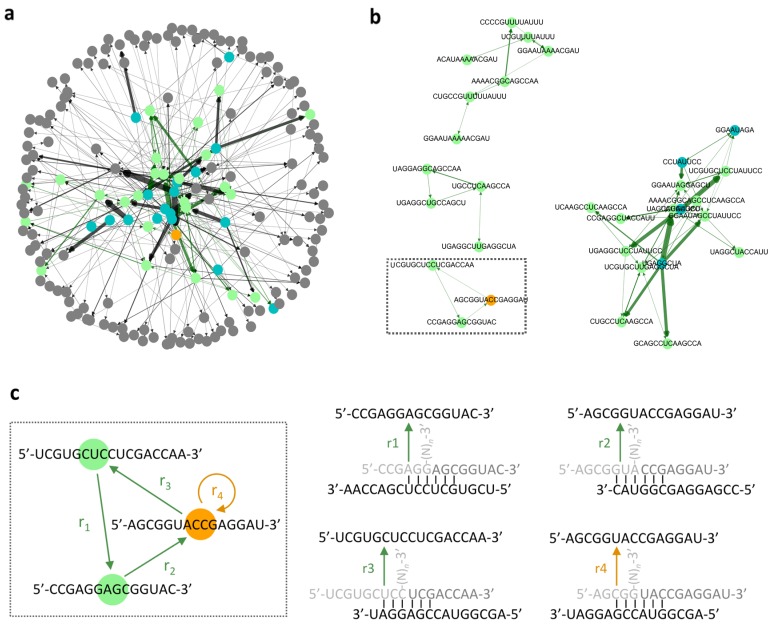
An example of a directed graph. (**a**) All of the analyzed reactions. Each node (circle) represents an RNA in the original population (blue), an RNA that self-reproduced (orange), an RNA in an autocatalytic network (green), and an RNA that was not categorized in any of them (gray). A former color was used if a node fell into two or more categories. Each edge (arrow) represents a catalytic reaction, from a catalyst toward a product. The width of an edge is proportional to the frequency of each reaction. A collectively autocatalytic reaction is highlighted in green. (**b**) Reproducing RNAs in panel (**a**) are featured. (**c**) The three-component cycle in panel (**b**) is highlighted (enclosed in a dotted rectangle). All the template-directed recombination reactions (r_1_, r_2_, r_3_, and r_4_) constituting the network were detailed. The light and medium grey sequences are substrates, in which (N)*_n_* represents arbitrary nucleotides with length *n* (≥1). When a reaction could occur through multiple combination of base pairs, only one example was shown. The simulation was performed with 300*X* initial RNAs and *X* = 20 for 30,000*X* reaction steps. The maximum network size in panel (**b**) was 11.

To understand whether reproducing RNAs emerge by iterative template-directed recombination over the simulation (Figure 1), we introduced a directed graph, an established method, to represent autocatalytic networks [24]. In a directed graph emerging in this study, each node represents the sequence of a catalyst (template of a reaction) or a product, and each edge represents a catalytic reaction, connecting from a catalyst to a product (Figure 2a). From the graph, we obtained collectively autocatalytic sets (green nodes and edges), where the synthesis of each RNA was catalyzed by at least one of the other members in a closed network, resulting in collective reproduction (Figure 2b). We also obtained self-reproducers (orange nodes) that catalyzed their own synthesis (edges not shown). These may also be involved in autocatalytic networks, but not necessarily. Figure 2c showed an example of RNA sequences and template-directed recombination reactions constituting a collectively autocatalytic set (a three-component cycle). 

### 3.2. The Highest Chance of Reproduction is at Intermediate Sequence Diversity within A Population

**Figure 3:**
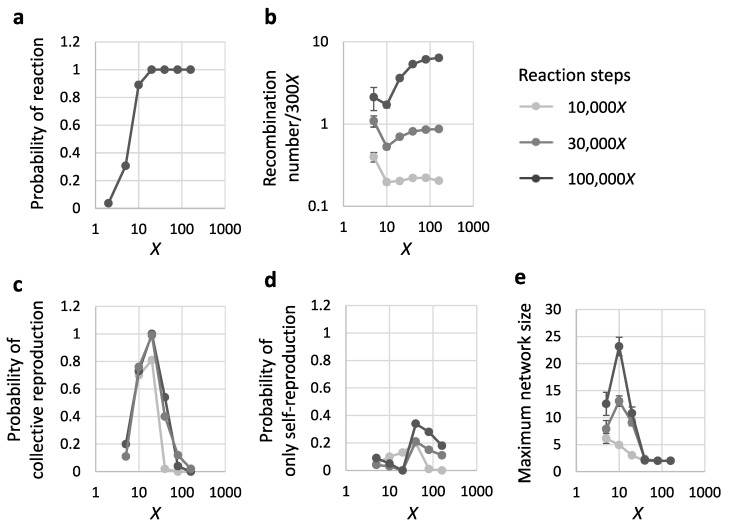
RNA reproduction by iterative template-directed recombination. The simulation was repeated 100 times with 300*X* initial RNAs for 10,000*X*, 30,000*X*, and 100,000*X* reaction steps. Each value was plotted against different diversities of an initial RNA population *X*. (**a**) Probability that at least one recombination occurred during simulation. (**b**) Average recombination number per population size (300*X*) when recombination occurs. (**c**) Probability that at least one autocatalytic network formed and collective reproduction occurred during a simulation. (**d**) Probability that no autocatalytic network formed, but self-reproduction of an RNA occurred. (**e**) Maximum network size of emerged autocatalytic networks. The error bars indicate standard errors in all panels. The data of *X* = 2 was omitted from panels (**b**–**e**) because the sample size was insufficient due to the low probability of reaction; see panel (**a**).

To investigate the possibility of the initiation of reproduction, we first performed the simulation with different sequence diversity *X* in a starting population of 300*X* 8-mer RNA sequences. *X* represents the number of distinct sequences in the population, and the multiplicity of each distinct sequence was fixed (300). We increased the number of reaction steps as the population size increased, and performed 10,000*X*, 30,000*X*, or 100,000*X* steps for each simulation. We repeated each simulation with a fixed parameter 100 times. Not surprisingly, if *X* was too low (*X* = 2 or 5), in most cases, recombination did not occur at all in the first place, hence reproduction could not be initiated (Figure 3a). Once recombination did occur, the number of recombination reactions relative to the population size was comparable for different *X* (Figure 3b). However, if *X* was too high (*X* = 80 or 160), for simulations with the three different number of reaction steps, although recombination occurred many times, autocatalytic networks or self-reproducers rarely emerged (Figure 3c,d). Critically, we found that reproduction can occur with a high probability (>80%), as either collective or self-reproduction, only in the intermediate range of sequence diversity (*X* = 10–20). At these diversities, reproduction occurred even after a small number of reaction steps (10,000*X*). In most of these cases, RNAs formed an autocatalytic network, and reproduced collectively. We also analyzed the number of distinct catalysts in the largest network as an indication of complexity. This number increased over reactions in the investigated parameters, reaching at most 23 on average for *X* = 10 (Figure 3e). These results suggest that the complex form of reproduction could begin even from an RNA population with a fairly modest amount of variation. 

### 3.3. There Are Too Many Unrelated Recombination Reactions at High Sequence Diversity within a Population

**Figure 4:**
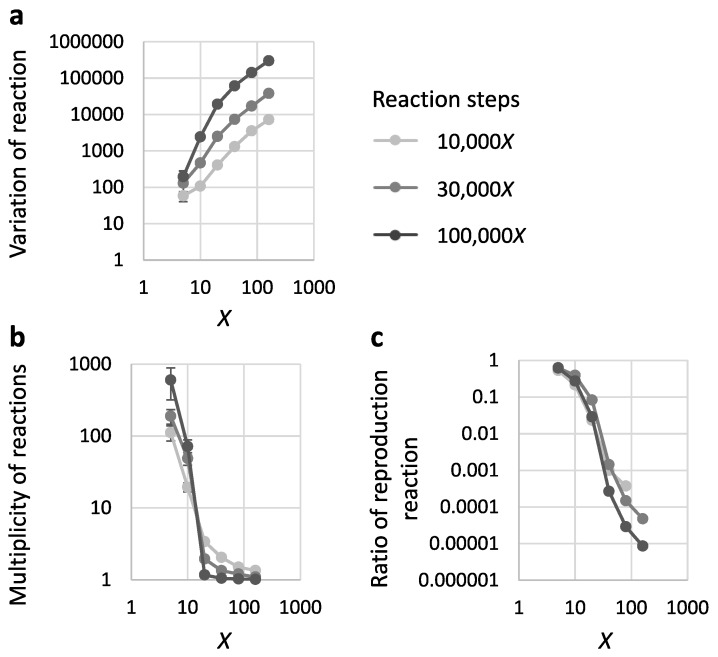
Decreased multiplicity of reactions at increased sequence diversity within a population. The simulation was repeated 100 times with 300X initial RNAs for 10,000X, 30,000X, and 100,000X reaction steps. Each value was plotted against different diversities of an initial RNA population, X. (**a**) The variation of reactions during a simulation. (**b**) Average multiplicity of reactions during a simulation. (**c**) Average ratio of reproduction reactions among all the reactions during a simulation in which reproduction occurred. In all panels, the error bars indicate standard errors.

To understand the mechanism by which the onset of reproduction was repressed at high sequence diversity in a given population, we analyzed the variation of recombination reactions (combination of a catalyst and a product) during the simulations. The average variation of reactions increased as the starting materials became more diverse because the number of possible combinations of recombinable RNAs also increased (Figure 4a). As a result, the multiplicity of reactions, defined as recombination numbers relative to the variation of reactions, decreased as the initial diversity *X* increased and reached nearly 1.0 at a high diversity (Figure 4b). Therefore, most of the reactions were novel, and consequently, almost no reproduction was possible. Consistently, when reproduction occurred in a simulation, the ratio of reproduction reactions to all the reactions decreased as *X* increased (Figure 4c), confirming the increase in unrelated recombination at high sequence diversity.

### 3.4. The Effect of Population Size on the Range of Intermediate Diversity

**Figure 5:**
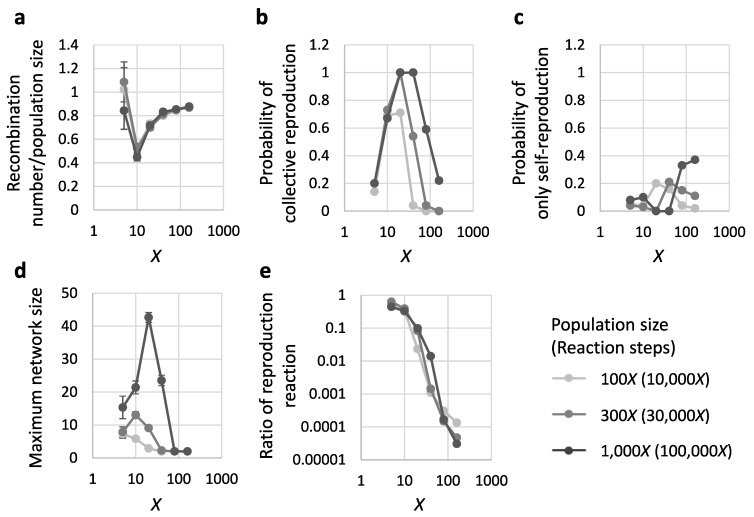
The simulation was repeated 100 times for 10,000*X*, 30,000*X*, and 100,000*X* reaction steps, with varied population sizes: 100*X*, 300*X*, and 1000*X*. Each value was plotted against different diversities of an initial RNA population *X*. (**a**) Average recombination number per population size when recombination occurs. (**b**) Probability that at least one autocatalytic network formed and collective reproduction occurred during a simulation. (**c**) Probability that no autocatalytic networks formed, but self-reproduction of an RNA occurred. (**d**) Maximum network size of emerged autocatalytic networks. The error bars indicate standard errors in all panels.

We next explored a factor that could affect the range of intermediate diversity from which reproduction is highly possible. We performed the simulation for the same set of initial sequence diversity in a given population *X* with three different population sizes: 100*X*, 300*X*, and 1,000*X*. We set 10,000*X*, 30,000*X*, and 100,000*X* reaction steps for each population size, respectively, so that the number of recombinations per sequence is comparable among different population sizes (Figure 5a). We found that, while the probability of (self- or collective) reproduction at a lower sequence diversity (*X* = 5–20) did not significantly change depending on the population sizes, the probability of reproduction at higher sequence diversity (*X* = 40–160) was improved as the population size increased (Figure 5b,c). At the largest population size (1000*X*) tested, a complex autocatalytic network emerged even at a relatively higher diversity *X* = 40 (Figure 5d), where the ratio of reproducing molecules in the populations was also improved (Figure 5e). These results imply that the optimal sequence diversity within a population for the reproduction could broaden toward higher diversity to some extent in a large population of RNA molecules.

### 3.5. Comparison of Template-Directed Recombination with Template-Directed Ligation

**Figure 6:**
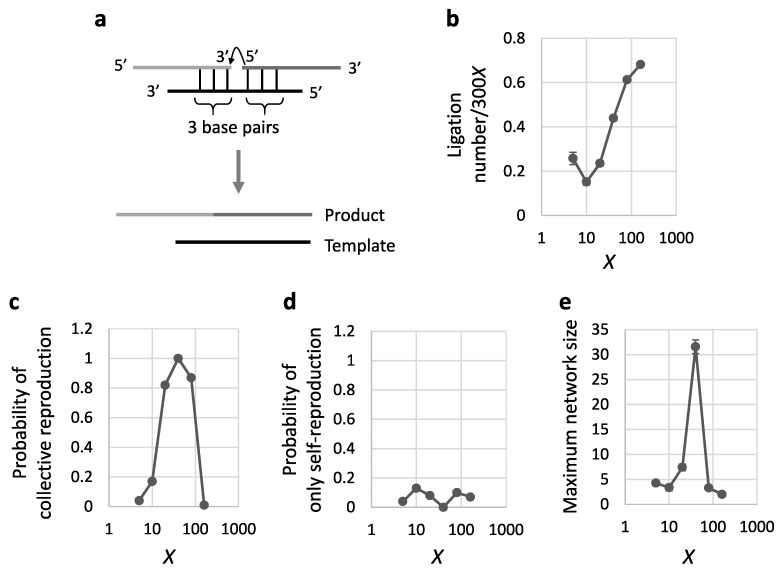
Initiation of RNA reproduction by iterative template-directed ligation. The simulation was repeated 100 times with 300*X* initial RNAs for 100,000*X* reaction steps. Each value was plotted against different diversities of an initial RNA population X. (**a**) Schematic representation of the template-directed ligation reaction. (**b**) Average ligation number per population size (300*X*) when ligation occurs. (**c**) Probability that at least one autocatalytic network formed and collective reproduction occurred during a simulation. (**d**) Probability that no autocatalytic network formed but self-reproduction of an RNA occurred. (**e**) Maximum network size of emerged autocatalytic networks. In all panels, the error bars indicate standard errors.

As a contrast to template-directed recombination, we also simulated template-directed ligation. Such ligation is another possible mechanism of prebiotic RNA reproduction, producing a longer sequence by conjugating two substrates if they form base-pairs with a template (Figure 6a), but some manner of condensation chemistry (e.g., a high-energy leaving group) is required for ligation. By the 100,000*X* reaction steps of template-directed ligation with an initial population of 300*X* 8-mer RNAs, we found similar trends to those of the template-directed recombination as described above, although the number of reactions was lower than that of recombination for the same number of reaction steps (Figure 3b and Figure 6b), probably because the possible combinations of substrates and a template were decreased. Similar to what was observed in template-directed recombination (Figure 3c,d), there was an intermediate range of diversity for an initial population that allowed reproduction by template-directed ligation (Figure 6c,d). However, as compared to recombination, the range in ligation reactions was slightly shifted toward higher diversities. Similar to the case of template-directed recombination (Figure 3e), reproducing RNAs in the intermediate range tended to form autocatalytic networks, with the largest network size 32 on average (*X* = 40) (Figure 6e), suggesting that the complex form of reproduction could begin from a modestly diverse population by template-directed ligation as well.

## 4. Discussion

The origin of reproducing information-carrying polymers is one of the long-standing (but heretofore unknown) phenomena important for the emergence of life. Here, we constructed and analyzed an RNA-based catalytic reaction model under prebiotically possible template-directed recombination and ligation. These modes of reproduction would have been more accessible to emergent polymeric pre-life than template-directed replication [17,18]. Using this system, we showed that at least in a subset of RNA population, sequence diversity within the population is a key factor to trigger RNA reproduction through iterative reactions. Reproduction was hindered in both low and high initial RNA diversity because of a too low or too large variety of possible reactions. Yet, we found that there was an intermediate range of diversity from which RNAs robustly started reproduction (Figure 3c,d and Figure 6c,d). The range was slightly different depending on the type of catalytic reaction, but was commonly seen in both template-directed recombination and ligation analyzed in this study. These results support the emergence of reproducing RNAs in prebiotic environments, where available RNA sequences were neither abundant nor diverse. 

Emergent reproducing RNAs at the intermediate sequence diversity tended to form autocatalytic networks and cooperatively catalyzed each other’s reproduction (Figure 3c, Figure 5b and Figure 6c). Furthermore, the size of emergent autocatalytic networks could increase over reactions to include at least dozens of catalysts for the investigated parameters (Figure 3e and Figure 5d), showing easy expansion of genetic informational capacity through primordial template-assisted reactions. These results portend a high potential of a complex autocatalytic network to spontaneously arise from small RNA molecules, supporting the plausibility of the formation of mutually catalytic molecular networks during abiogenesis [21,23,31]. However, it should be noted that we did not investigate whether or not obtained autocatalytic networks were completely closed in the sense that no unrelated reaction was catalyzed by any of the members. Because the variety of reactions in a simulation was considerable (Figure 4), it is highly possible that there were some unrelated reactions coming off the networks, which should have diminished their strength of autocatalysis, although reproduction still occurred.

One may argue that the actual diversity of RNA sequences on the early Earth could have been higher than the range investigated in the study (*X* = 2–160). Although it was difficult to examine a much higher diversity on our simulation as a consequence of finite computational resources, the expansion of the optimal diversity depending on the population size (i.e., concentration) suggests that reproduction could have also started from a highly diverse concentrated RNA population. It should be noted, however, that we focused on only one type of reaction (a template-directed recombination or ligation) in a given simulation, and did not consider other reactions (e.g., hydrolysis and other template-directed reactions). If multiple different reactions happened simultaneously, it would generate more unrelated or potentially interfering RNAs, making reproduction increasingly difficult. In such a chaotic milieu, the incorporation of new factors such as sequence specificity of template-directed recombination and ligation, if any, may be required to achieve reproduction. 

To focus on the initiation of reproduction, we investigated the reproduction only in a relatively short-time scale. Important future work therefore should include the simulation of long-term sustainability and remodeling (evolvability) of autocatalytic networks through continuous supply of resources and degradation or dilution, valuable aspects for the propagation of genetic information [21,24]. This was previously performed for an abstract chemical system [24]. Regarding sustainability, although not systematically investigated, one result we did find in our study was that an emergent autocatalytic network sometimes incorporated originally extant short RNA resources (Figure 2b), implying that the autocatalytic RNA sets re-generated useful resources for reproduction. To date, there have been only a few studies on such an RNA property of recycling materials, or catabolism in a more general sense [32,33]. Our simulation constructed here could reveal favorable conditions to initiate self-sustained RNA reproduction through processing useless materials in a future study.

## 5. Conclusions

In this study, we demonstrated that a subset of short RNAs with limited diversity had potential to kick-start autocatalytic reproduction thorough successive primordial recombination and ligation. The dominant mode of reproduction was an autocatalytic network, supporting the plausibility of cooperation among abiotic RNAs. These results indicate that the limited resources possibly available in prebiotic environments may have been sufficient for the foundation of reproduction, a key living process for the origins of life, both on the Earth and elsewhere in the universe. Although our simulation presented here only focused on the initiation of reproduction, further simulations could contribute in future to characterizing sustainability and evolvability of reproducing RNA sets, and if combined, key parameters for gradual steps toward life-like reproduction during abiogenesis would be unlocked. 

## Data Availability

The codes for the simulations are available at GitHub (https://git.io/fhFIG).
